# Bioremediation by *Cupriavidus metallidurans* Strain MSR33 of Mercury-Polluted Agricultural Soil in a Rotary Drum Bioreactor and Its Effects on Nitrogen Cycle Microorganisms

**DOI:** 10.3390/microorganisms8121952

**Published:** 2020-12-09

**Authors:** Guillermo Bravo, Paulina Vega-Celedón, Juan Carlos Gentina, Michael Seeger

**Affiliations:** 1 Molecular Microbiology and Environmental Biotechnology Laboratory, Department of Chemistry & Center of Biotechnology Daniel Alkalay Lowitt, Universidad Técnica Federico Santa María, Avenida España 1680, Valparaíso 2390123, Chile; pvegaceledon@gmail.com; 2 School of Biochemical Engineering, Pontificia Universidad Católica de Valparaíso, Avenida Brasil 2085, Valparaíso 2362803, Chile; carlos.gentina@pucv.cl

**Keywords:** *Cupriavidus metallidurans*, mercury, nitrogen cycle, rotary drum bioreactor, soil bioremediation

## Abstract

Nitrogen cycle microorganisms are essential in agricultural soils and may be affected by mercury pollution. The aims of this study are to evaluate the bioremediation of mercury-polluted agricultural soil using *Cupriavidus metallidurans* MSR33 in a rotary drum bioreactor (RDB) and to characterize the effects of mercury pollution and bioremediation on nitrogen cycle microorganisms. An agricultural soil was contaminated with mercury (II) (20–30 ppm) and subjected to bioremediation using strain MSR33 in a custom-made RDB. The effects of mercury and bioremediation on nitrogen cycle microorganisms were studied by qPCR. Bioremediation in the RDB removed 82% mercury. MSR33 cell concentrations, thioglycolate, and mercury concentrations influence mercury removal. Mercury pollution strongly decreased nitrogen-fixing and nitrifying bacterial communities in agricultural soils. Notably, after soil bioremediation process nitrogen-fixing and nitrifying bacteria significantly increased. Diverse mercury-tolerant strains were isolated from the bioremediated soil. The isolates *Glutamicibacter* sp. SB1a, *Brevundimonas* sp. SB3b, and *Ochrobactrum* sp. SB4b possessed the *merG* gene associated with the plasmid pTP6, suggesting the horizontal transfer of this plasmid to native gram-positive and gram-negative bacteria. Bioremediation by strain MSR33 in an RDB is an attractive and innovative technology for the clean-up of mercury-polluted agricultural soils and the recovery of nitrogen cycle microbial communities.

## 1. Introduction

Lands for agriculture are degraded due to pollution and other anthropogenic activities [1]. Anthropogenic activities have significantly perturbed the soil nitrogen cycle [2,3,4,5]. Nitrogen is an essential nutrient for all forms of life and a key element in the agroecological system [6,7]. Nitrogen cycle balance in soils is influenced principally by microbial nitrogen fixation, nitrification, and denitrification [8]. In agricultural soils, nitrogen-fixing bacteria is an indicator of soil quality [7,9]. Nitrogen-fixing microorganisms contribute to plants productivity and are essential in agricultural systems [6,10,11]. Nitrification is performed by bacteria and archaea, which oxidize ammonia to nitrite and nitrate [2,8]. These conversions could affect agricultural soil, due to losses generated by the high solubility of nitrate that may cause groundwater eutrophication. Denitrification is a process in which nitrate is reduced successively to NO, N_2_O, and N_2_ gases, and may affect agricultural soil due to nitrogen release into the atmosphere [5,8,12].

The presence of heavy metals in the environment harms ecosystems, affecting agricultural soil quality and human health [13]. Mercury pollution of agricultural soil is caused mainly by mercurial compounds present in pesticides, seed-coat dressing, mercury-polluted manures, disinfectants, pharmaceuticals, and the mobilization of metal-containing sludge [13,14,15,16]. Mercury is a toxic heavy metal that causes deleterious effects on organisms due to its high affinity to sulfhydryl and thioester groups on proteins [17,18]. Mercury causes growth arrest, oxidative stress, disruption of the integrity of cell membranes, interference with the electron transport system, enzymatic inhibition, and replacement of metallic centers of metalloproteins [19,20,21,22]. Heavy metal pollution causes major changes in soil microbial composition and their activities [3,13,23]. In plants, mercury replaces the central magnesium atom of chlorophyll, interrupting photosynthesis, and decreasing photosynthetic pigments, causing damage to agricultural systems [14,24].

Bioremediation is an eco-friendly and low-cost treatment based on the capability of bacteria, fungi, archaea, and plants to remove or transform compounds or elements into less toxic forms [22,25,26,27,28]. Mercury bioremediation by mercury-resistant bacteria is based on the reduction of Hg (II) to gaseous Hg (0) by proteins encoded by the *mer* genes [16,19,29,30,31,32,33]. *Cupriavidus metallidurans* is a facultative anaerobic bacterium capable of removing heavy metals (Hg, Cd, Cu) and degrading toxic organic pollutants such as toluene under aerobic and anaerobic conditions [19,22,34,35,36,37]. *C. metallidurans* strain MSR33 is a transconjugant derivative of *C. metallidurans* CH34, which contains the environmental plasmid pTP6 [19]. Strain MSR33 possesses an increased mercury resistance (2.4-fold), reducing Hg (II) and organomercurial compounds into Hg (0) under aerobic and anaerobic conditions [19,22]. A method for the bioremediation of environments polluted with mercury, copper, and cadmium using *C. metallidurans* strain MSR33 has been patented [38]. Several processes for mercury bioremediation from aqueous solutions have been described [19,22,29,39,40]. However, the bioremediation of mercury-polluted soil has been scarcely studied. Bioaugmentation using zeolite immobilized *Pseudomonas veronii* of mine tailing soil polluted with Hg (II) (7 ppm) increases 4-fold the background mercury volatilization [31]. Bioremediation using the fungus *Lecythophora* sp. DC-F1 and biochar of mercury-polluted soil (30 ppm) reported 13.3–26.1% mercury removal after 56 days [41]. Phytoremediation using *Triticum aestivum* showed 70% mercury removal in mercury-polluted soil (~30 ppm) after 3 years [42]. The hybrid plant *Miscanthus* × *giganteus* showed a mercury removal rate of 4 µg year^−1^ in Hg-polluted soil (20 ppm) [43].

Rotary drums are used in industrial processes of drying, incineration, humidification, mixing of solid particles, and biological applications, such as microbial biomass production and soil bioremediation [44,45,46,47,48,49]. Rotary drum bioreactor (RDB) is an attractive alternative for ex situ soil bioremediation due to its absence of internal moving parts for mixing, simple construction, simple operation, and reduced aeration costs [47,48]. The soil bioremediation of fluorene, anthracene, phenanthrene, pyrene, toluene, diethyl ether, hexane, synthetic dyes, and petroleum hydrocarbon in RDB were described [44,45,46,50,51]. Bioleaching processes of metals on RDB were described for gold, copper, zinc, and nickel ores [52].

The aims of this study are to evaluate the bioremediation of mercury-polluted agricultural soil using *Cupriavidus metallidurans* MSR33 in a rotary drum bioreactor (RDB) and to characterize the effects of mercury pollution and bioremediation on nitrogen cycle microorganisms. The mercury bioremediation in a custom-made RDB was performed. The effects of MSR33 cell concentrations (6 and 3 g cells kg^−1^ dry soil), thioglycolate (5 mM), and Hg (II) concentrations (20 and 30 ppm) on mercury soil removal were studied. Mercury showed a negative effect on nitrogen-fixing and nitrifying bacterial communities in agricultural soils. In contrast, after bioremediation by strain MSR33, nitrogen-fixing and nitrifying bacterial communities significantly increased. Six mercury-tolerant strains were isolated from bioremediated soils. The isolates were identified as *Glutamicibacter* sp. SB1a, *Bacillus* sp. SB1b, *Planomicrobium* sp. SB2b, *Bergeyella* sp. SB2a, *Brevundimonas* sp. SB3b, and *Ochrobactrum* sp. SB4b. *Glutamicibacter* sp. SB1a, *Brevundimonas* sp. SB3b, and *Ochrobactrum* sp. SB4b possess the *merG* gene that is associated with the plasmid pTP6, suggesting the horizontal transfer of this plasmid from *C. metallidurans* strain MSR33 to native gram-positive and gram-negative soil bacteria.

## 2. Materials and Methods

### 2.1. Chemicals

Succinate, HgCl_2_, sodium thioglycolate, H_2_SO_4_, NH_4_Cl, NaH_2_PO_4_ × 2H_2_O, KCl, HCl, NaOH, agarose, and KAPA SYBR FAST qPCR Master Mix were purchased from Merck (Darmstadt, Germany). Primers (Table 1) were purchased from IDT (Coralville, IA, USA). FastDNA Spin Kit for soil and GeneClean II Spin Kit were purchased from MP Biomedicals (Solon, OH, USA). GoTaq Green Master Mix was purchased from Promega (Madison, WI, USA). GelRed Nucleic Acid Gel Stain was purchased from Biotium (Fremont, CA, USA). Cycloheximide was purchased from USBiological (Salem, MA, USA).

### 2.2. Strains

*Cupriavidus metallidurans* MSR33 (positive control for the *zniA* and *merG* genes) and *Paraburkholderia xenovorans* LB400 (positive control for the *nifH* gene) were obtained from the culture collection of Molecular Microbiology and Environmental Biotechnology Laboratory, Universidad Técnica Federico Santa María (Valparaíso, Chile). *Escherichia coli* clone AOB amoA (positive control for the AOB *amoA* gene) was kindly provided by Julieta Orlando, Faculty of Sciences, Universidad de Chile (Santiago, Chile).

### 2.3. Agricultural Soil Samples

Non-polluted agricultural sandy loam soil samples were collected at Casablanca valley, Central Chile in March 2016, as described by Altimira et al. [3]. The non-polluted site was located in La Vinilla (longitude 71°24′36′′ W and latitude 32°19′30.254′′ S). Previous soil analysis determined 2.3% organic matter content, low heavy metal content, and neutral pH [3]. Soil samples were air-dried, 2 mm sieved, and homogenized. The soil samples were stored in polyethylene bags and preserved in a dark room at 4 °C until analyses.

### 2.4. Preparation of Mercury-Polluted Agricultural Soil

Agricultural soil was spiked with HgCl_2_ solutions to obtain mercury-polluted soil (20 and 30 ppm). The soil suspension was homogenized with a ceramic mortar, dried for 7 days at 30 °C, and crushed to recover the original granulometry (2 mm). Subsequently, mercury content in soil was determined by atomic absorption spectrometry [22].

### 2.5. Batch Culture Growth

Batch culture growth of strain MSR33 was performed in a stirred-tank bioreactor Ez-control (Applikon Biotechnology, Delft, The Netherlands) of 3 L total volume, equipped with a Rushton type turbine and pH and temperature controllers. MSR33 cells were grown in GBC medium (pH 7) with succinate (8 g L^−1^) in 1 L fermentation volume with agitation (500 rpm), aeration (airflow of 2 vvm) and at 30 °C [22]. Previously, MSR33 cells grown in Luria–Bertani broth medium (until late exponential phase) were harvested and inoculated at 10% *v*/*v* in the fermentation volume. The bacterial cultures were collected at early stationary phase for the inoculation in soil during bioremediation assays.

### 2.6. Bioremediation of Mercury-Polluted Agricultural Soils in a Rotary Drum Bioreactor (RDB)

**Figure 1:**
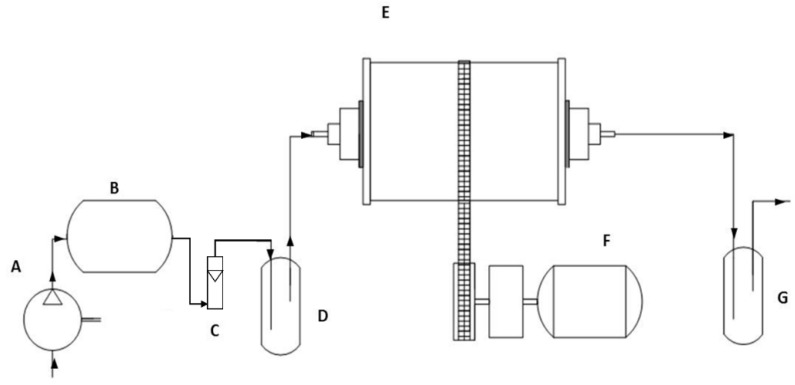
Flow chart of the designed and custom-made rotary drum bioreactor (RDB) for the bioremediation of mercury-polluted agricultural soils. (**A**) Air compressor; (**B**) Air accumulator balloon; (**C**) Air rotameter; (**D**) Air humidifier; (**E**) RDB; (**F**) Rotation mechanism; (**G**) Mercury gas oxidizing trap containing HNO_3_ (1 M). Arrows indicate gas flow during operation. Hg (II) in polluted soil is reduced to Hg (0) gaseous by *C. metallidurans* MSR33 in the RDB (**E**). Gas is displaced from the RDB by air injection (**A**–**D**). Gaseous Hg (0) is captured by gas stripping in an oxidizing trap, where Hg (0) is oxidized to Hg (II) (**G**).

An acrylic RDB of 20 L and 8 internal lifters (5 cm width), equipped with a humidified air injection system and mercury gas oxidizing trap (HNO_3_ 1M), was designed, and built. The RDB was used for bioremediation treatments using *C. metallidurans* MSR33 of mercury-polluted soils (Figure 1). Agricultural soil bioremediation treatments were performed in a thermo-regulated chamber (30 ± 3 °C), a constant drum rotation speed of 10 rpm, and a humidified air injection of 1 vvm. All treatments were evaluated by mixing a cell-culture suspension and soil in a 2:1 ratio. 500 g of mercury-polluted soils and 1 L cell culture of strain MSR33 were mixed in the RDB during soil bioremediation assay. Negative controls were performed using sterile distilled water and soil in a 2:1 ratio. For each treatment, samples in duplicate were analysed.

The effect of cell concentration on mercury bioremediation was evaluated during 72 h using 6 and 3 g cells kg^−1^ dry soil, and mercury-polluted soils (20 ppm).

The effect of thioglycolate application on mercury bioremediation was evaluated using 6 g cells kg^−1^ dry soil, and mercury-polluted soils (20 ppm). Thioglycolate (5 mM) was added at the beginning of the assay. Soil bioremediation assays were evaluated during 96 h.

The re-inoculation assay was conducted using 6 g cells kg^−1^ dry soil and mercury-polluted soils (20 ppm). At 48 h after the bioremediation started, MSR33 cells (6 g cells kg^−1^ dry soil) were inoculated in the RDB. MSR33 cells from 1 L batch culture were collected in a Hettich model Rotina 380R centrifuge (Kirchlengern, Germany) at 3500× *g* for 10 min. Soil bioremediation assays were run during 96 h.

The effect of mercury content in soil on bioremediation was evaluated during 72 h using 6 g cells kg^−1^ dry soil and mercury-polluted soils (20 and 30 ppm).

### 2.7. Soil Mercury Determination

For the determination of Hg in soil samples (1.5 g), the AOAC 977.15 methodology was used with modifications [56]. The mercury quantification was carried out by cold vapor atomic absorption spectrometry using an atomic absorption spectrometer Agilent model 240AA series AA1110M032 (Santa Clara, CA, USA), with a hydride generation module (VGA 77) [22].

### 2.8. Bacterial Count and Isolation of Mercury-Tolerant Strains

Heterotrophic bacteria and mercury-tolerant bacteria in soil were quantified. Each moist soil sample (1 g) was diluted in 9 mL of PBS (phosphate-buffered saline). The dilution was mixed vigorously and subjected to orbital agitation of 200 rpm for 30 min. Serial dilutions of the suspensions were grown in TSA in the absence or presence of mercury (II) (10 ppm) [3]. To prevent fungal growth, cycloheximide (100 µg L^−1^) was added to the culture medium [57]. The plates were incubated at 30 °C. Heterotrophic and mercury-tolerant cultivable bacteria were counted after 72 h. To determine the CFU g^−1^ dry soil, the soil moisture was determined on a Moisture Analyzer Sartorius MA 35 (Göttingen, Germany). MSR33 colonies were determined based on their capability to grow in the presence of Hg (II) (10 ppm). Novel mercury-tolerant bacterial strains were isolated and identified.

### 2.9. Genomic DNA Extraction from Bacterial Isolates and PCR Amplification of 16S rRNA and merG Genes

Genomic DNA was prepared from single colonies suspended in 100 μL of sterile milli-Q water, heated at 95 °C for 5 min, and centrifuged briefly [3]. The supernatant (2 μL) was used for PCR amplification. PCR reactions were conducted in a volume of 25 μL containing specific primers (0.6 μM each), GoTaq Master Mix reagent (12.5 μL), and 1 μL genomic DNA. The forward 27F and reverse 1492R primers were used for the 16S rRNA gene amplification (Table 1). The forward merG_F and reverse merG_R primers were used for the *merG* gene amplification (Table 1).

DNA amplification of the 16S rRNA gene was carried out using the following conditions: 1 cycle of 95 °C for 5 min, 30 cycles of 95 °C for 1 min, 55 °C for 1 min, 72 °C for 1.5 min, and a final extension of 72 °C for 10 min. Amplification of the *merG* gene was carried out using the following conditions: 1 cycle of 95 °C for 3 min, 35 cycles of 95 °C for 30 s, 55 °C for 30 s, 72 °C for 30 s, and a final extension of 72 °C for 5 min. *C. metallidurans* MSR33 genomic DNA was used as a positive control for the *merG* gene. PCR products were visualized by agarose gel electrophoresis (2% *w*/*v*), followed by staining with GelRed Nucleic Acid Gel Stain (1:10,000 *v*/*v*).

### 2.10. 16S rRNA Gene Sequence Analyses

The 16S rRNA gene amplification products were quantified using the Qubit fluorometer (Invitrogen, Carlsbad, CA, USA). The partial 16S rRNA gene PCR products were sequenced using the primer 800R in Macrogen Inc. (Seoul, Korea). The sequences were compared with the Genbank NCBI databases to determine the genera of the bacterial strains. A phylogenetic analysis was performed to study the evolutionary relationships of the sequences based on the alignments calculated by CLUSTAL W using the default options. The evolutionary history was inferred using the neighbor-joining method. Evolutionary analyses were conducted in MEGA 5.2.2 software [58]. The 16S rRNA gene sequences were deposited in GenBank under the following accession numbers: *Glutamicibacter* sp. SB1a (MT953323), *Bacillus* sp. SB1b (MT953319), *Planomicrobium* sp. SB2b (MT953320), *Bergeyella* sp. SB2a (MT953324), *Brevundimonas* sp. SB3b (MT953321), and *Ochrobactrum* sp. SB4b (MT953322).

### 2.11. Metagenomic DNA Extraction from Agricultural Soil

Metagenomic DNA from agricultural soils was extracted. Total soil DNA was extracted from 0.5 g of dry soil using the FastDNA Spin Kit for soil with mechanical lysis by two pulses at 5.5 m s^−1^ per 30 s in the FastPrep-24 Bead-Beater Instrument (MP Biomedicals, Solon, OH, USA). After DNA extraction from the soil, DNA was directly purified with the GeneClean II Spin Kit following the manufacturer’s instructions. Purified DNA samples were quantified using the Qubit fluorometer according to the manufacturer’s instructions.

### 2.12. Quantification of Nitrogen Cycle-Associated Bacteria and Strain MSR33 in Agricultural Soils

The standard-curve method of absolute quantification was used for the analysis of nitrogen cycle-associated *nifH* (nitrogen fixation) and AOB *amoA* (nitrification) genes, strain MSR33 *zniA* gene (chromid) and plasmid pTP6 *merG* gene. Agricultural soil metagenomic DNA samples were subjected to quantitative PCR (qPCR) of these genes.

Genomic DNA of *P. xenovorans* LB400 was used for the *nifH* gene detection, *E. coli* clone AOB *amoA* for the AOB *amoA* gene detection, and *C. metallidurans* MSR33 for the *zniA* and *merG* genes detection. The qPCR was done on a Stratagene Mx3000pTM (Agilent Technologies, Santa Clara, CA, USA), using the KAPA SYBR FAST qPCR Master Mix reagent and 0.2 µM from each primer (Table 1) following the manufacturer’s instructions. The amplification of a single PCR product for each pair of primers was confirmed by fusion curves.

The standard curves for each amplicon were performed in triplicate by serial dilutions (1:10) of genomic DNA from the positive controls of *nifH*, AOB *amoA*, *zniA*, and *merG* genes. The efficiency of the qPCR reaction for each gene was calculated from the slopes of the exponential portion of the calibration curves. The gene copy number per gram of soil was determined according to the methodology described by Brankatschk et al. [59]. Absolute qPCR of *nifH,* AOB *amoA*, *zniA* and *merG* genes were carried out using the following conditions: 1 cycle at 95 °C for 3 min, 40 cycles at 95 °C for 30 s, 55 °C for 30 s, 72 °C for 30 s, and a final extension at 72 °C for 5 min.

### 2.13. Statistical Analysis

The data were analysed by one-way ANOVA. After carrying out one-way ANOVA, Fisher’s LSD test was used to determine significant differences (*p* < 0.05) among the treatments.

## 3. Results

### 3.1. Evaluation of Operational Parameters on Bioremediation by Strain MSR33 of Mercury-Polluted Agricultural Soil in an RDB

**Figure 2:**
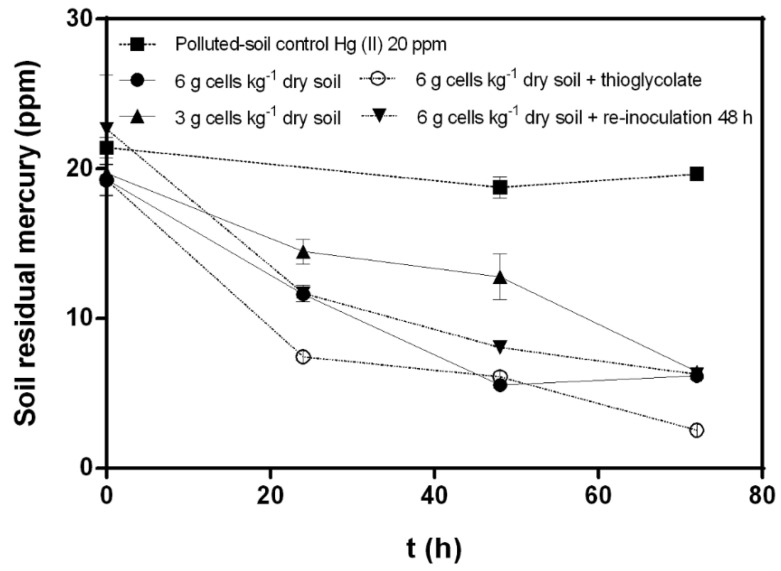
Effects of cellular concentration, thioglycolate, and cellular re-inoculation on mercury (II) (20 ppm) bioremediation by *C. metallidurans* MSR33 on mercury-polluted agricultural soil in a rotary drum bioreactor. (■) Hg-polluted soil control, (●) Hg-polluted soil bioremediation with MSR33 cells concentration of 6 g cells kg^−1^ dry soil, (▲) Hg-polluted soil bioremediation with MSR33 cells concentration of 3 g cells kg^−1^ dry soil, (○) Hg-polluted soil bioremediation with MSR33 cells concentration of 6 g cells kg^−1^ dry soil plus thioglycolate (5 mM), (▼) Hg-polluted soil bioremediation with MSR33 cells concentration of 6 g cells kg^−1^ dry soil plus cells re-inoculation at 48 h. The assays were performed in duplicate; bars indicate the standard deviation.

**Figure 3:**
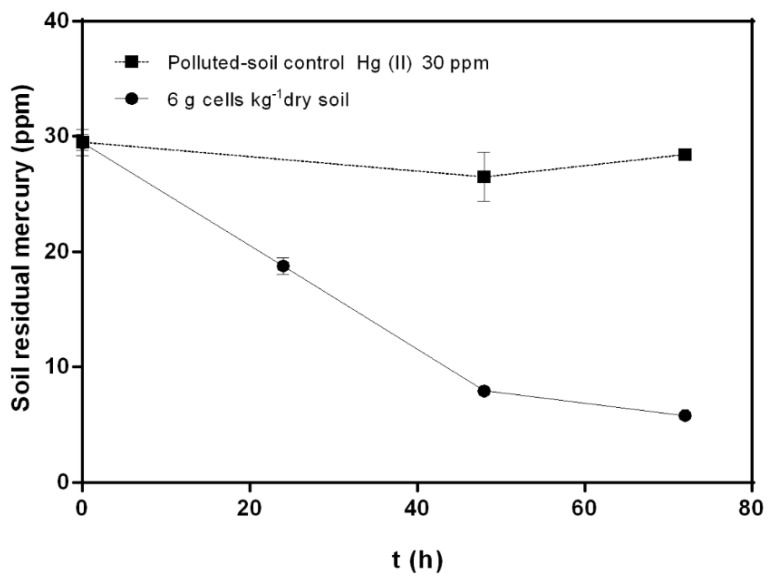
Effects of bioremediation by *C. metallidurans* MSR33 on agricultural soil polluted with mercury (II) (30 ppm) in rotary drum bioreactor. (■) Hg-polluted control soil, (●) Hg-polluted soil bioremediation using 6 g cells kg^−1^ dry soil MSR33 cells concentration. The assays were performed in duplicate. Bars indicate the standard deviation.

A bioremediation process of mercury-polluted soils with *C. metallidurans* strain MSR33 in a custom-made RDB was established. Mercury (II) removal kinetics variables: cellular concentration, thioglycolate, re-inoculation, and mercury concentration were evaluated (Figure 2 and Figure 3).

Mercury bioremediation with MSR33 cell concentrations of 6 and 3 g cells kg^−1^ dry soil were evaluated. Bioremediation performed with a high concentration of MSR33 cells (6 g cells kg^−1^ dry soil) showed a higher mercury removal after 48 h (70% mercury removal) than with 3 g cells kg^−1^ dry soil. However, after 72 h, both treatments reached a similar mercury residual concentration in soil (6.16 ± 0.06 ppm and 6.43 ± 0.08 ppm, respectively) (Figure 2).

The effect of thioglycolate on Hg (II) (20 ppm) removal was determined. Thioglycolate (5 mM) increased the mercury removal (82%), reaching a lower concentration of residual mercury (2.53 ± 0.014 ppm) in soil after 72 h, compared to mercury removal by treatment without thioglycolate (70%). The effect of re-inoculation on the mercury bioremediation process was also studied. A second inoculation with 6 g cells kg^−1^ dry soil at 48 h showed no changes in mercury removal from polluted soils compared with bioremediation treatments without re-inoculation. Re-inoculation treatment reached a residual mercury concentration of 6.27 ± 0.06 ppm after 72 h. The effect of a higher concentration of mercury (II) (30 ppm) on soil bioremediation by strain MSR33 in RDB was evaluated with 6 g cells kg^−1^ dry soil (Figure 3). Strain MSR33 removed 80% of mercury after 72 h, achieving a residual concentration of 5.81 ± 0.34 ppm mercury in soil. Similar removal kinetics behavior was observed in soils polluted with mercury 20 ppm (Figure 2).

### 3.2. Effects of Mercury and Mercury Bioremediation on Bacterial Communities of Agricultural Soils

The effects of mercury and mercury bioremediation by strain MSR33 on bacterial communities in agricultural soils were evaluated by plate counting of total heterotrophic bacteria and mercury-tolerant bacteria. Mercury-tolerant bacteria (10 ppm) were isolated and identified by 16S rRNA partial gene sequence analysis. The presence of the *merG* gene in the isolated strains was evaluated. The effects of mercury and mercury bioremediation on bacteria associated with the nitrogen cycle of agricultural soils and mercury tolerance were evaluated through the quantitative PCR of genes associated with nitrogen fixation, nitrification, strain MSR33 and plasmid pTP6.

**Figure 4:**
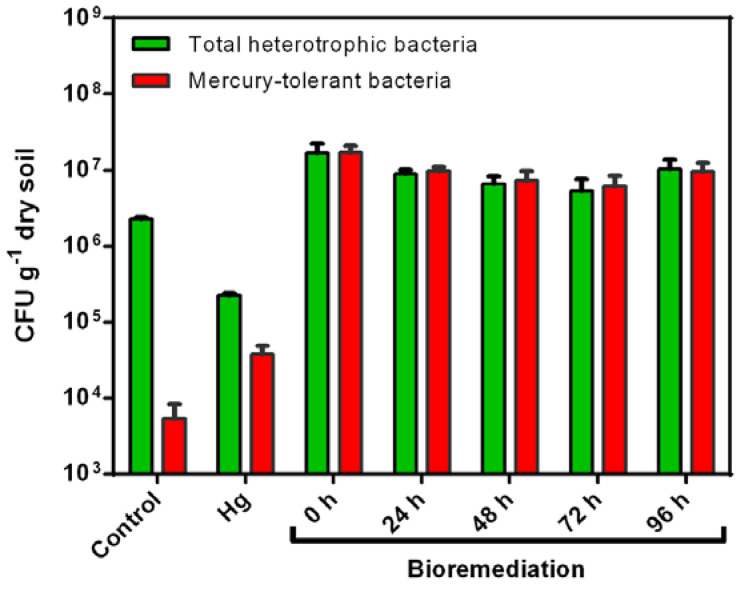
Effect of mercury and mercury bioremediation on the number of total heterotrophic and mercury-tolerant bacteria in agricultural soils. Total heterotrophic and mercury-tolerant bacteria were evaluated on TSA medium and TSA medium-plus mercury (10 ppm), respectively. Control: non-polluted agricultural soil; Hg: agricultural soil exposed to mercury (II) (20 ppm); Bioremediation: mercury-polluted agricultural soil bioremediated on rotary drum bioreactor by *C. metallidurans* strain MSR33 at different times. Green bars indicate total heterotrophic bacteria and red bars indicate mercury-tolerant bacteria. The bacterial count was performed in quintuplicate. Bars indicate the standard deviation.

Mercury-polluted soil showed a decrease in total heterotrophic bacteria and an increase in mercury-tolerant bacteria compared to non-polluted soil (Figure 4). The heterotrophic and mercury-tolerant bacteria were increased by the bioremediation process.

**Figure 5:**
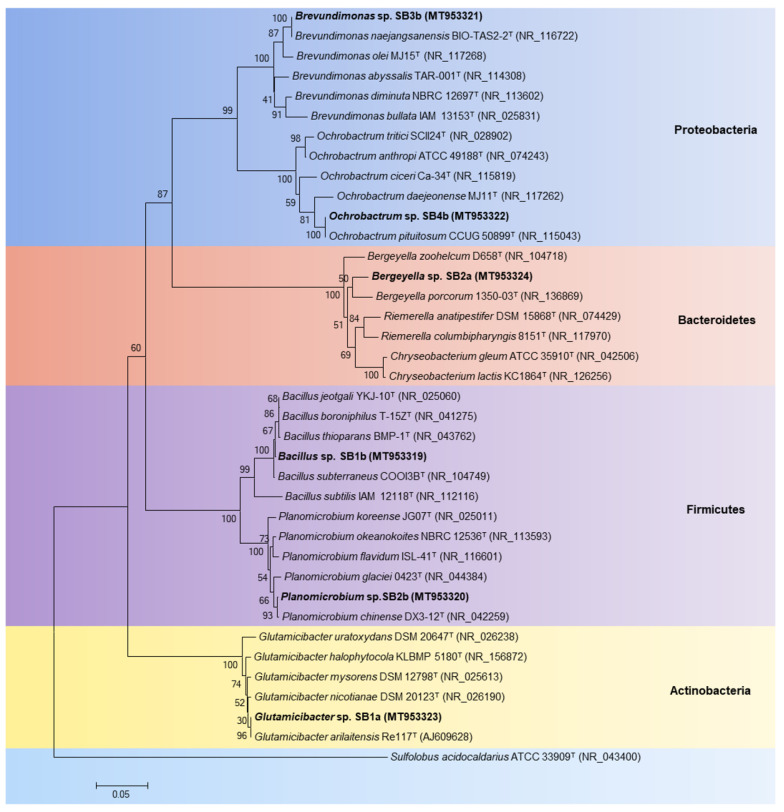
Identification by 16S rRNA gene sequence analyses of mercury-tolerant bacterial strains from bioremediated agricultural soil. The phylogenetic tree was constructed using Neighbor-joining method. The tree has arbitrarily been rooted by the archaeon *Sulfolobus acidocaldarius*. Values of 1000 bootstrap are informed at the branching point. GenBank accession numbers of 16S rRNA sequences are indicated in parentheses. Scale bar represents 0.05 substitutions per nucleotide positions.

For bacterial identification, comparative 16S rRNA gene (400–700 pb) sequence analyses of the isolates were performed. The results indicated that strain SB1a belongs to the *Glutamicibacter* genus of the Actinobacteria phylum, showing 100% 16S rRNA gene sequence similarity to the type strain *Glutamicibacter arilaitensis* Re117^T^. Strain SB1b was identified as a *Bacillus* member of the Firmicutes phylum, with 100% 16S rRNA gene sequence similarity to the type strain *Bacillus subterraneus* COOI3B^T^. The isolate SB2b was identified as a *Planomicrobium* strain of the Firmicutes phylum, showing 100% 16S rRNA gene sequence identity with the type strain *Planomicrobium chinense* DX3-12^T^. Strain SB2a was associated to the *Bergeyella* genus of the Bacteroidetes phylum, with 96% 16S rRNA gene sequence similarity with the type strain *Bergeyella porcorum* 1350-03^T^. Strain SB3b belongs to *Brevundimonas* genus of the Proteobacteria phylum, possessing 100% 16S rRNA gene sequence identity to the type strain *Brevundimonas naejangsanensis* BIO-TAS2-2^T^. The isolate SB4b belongs to the *Ochrobactrum* genus of the Proteobacteria phylum, with 100% 16S rRNA gene sequence similarity to the type strain *Ochrobactrum pituitosum* CCUG 50899^T^. The 16S rRNA gene sequences of the isolated strains and other reported bacteria, including type strains from *Glutamicibacter*, *Bacillus*, *Planomicrobium*, *Bergeyella*, *Brevundimonas* and *Ochrobactrum* genera, were used to build a phylogenetic tree (Figure 5).

**Figure 6:**
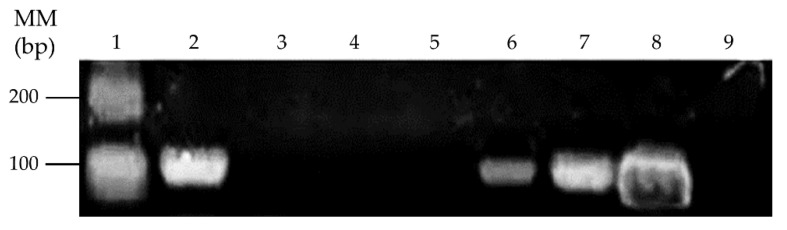
Detection of the *merG* gene in mercury-tolerant bacterial strains from bioremediated agricultural soil. Lane 1, Molecular mass marker 100 bp plus. Lanes 2–7, Mercury-tolerant bacterial strains SB1a, SB2a, SB1b, SB2b, SB3b, and SB4b. Lane 8, the *merG* gene positive control (*C. metallidurans* strain MSR33 genomic DNA). Lane 9, Negative control.

In order to determine if mercury tolerance of these strains may be associated to horizontal gene transfer of broad-spectrum mercury resistance genes from strain MSR33, the presence of the *merG* gene was evaluated by PCR in mercury-tolerant isolates (Figure 6).

The gram-positive strain *Glutamicibacter* sp. SB1a, and the gram-negative strains *Brevundimonas* sp. SB3b and *Ochrobactrum* sp. SB4b possess the *merG* gene. These results suggest the horizontal gene transfer of broad-spectrum mercury resistance genes from *C. metallidurans* strain MSR33 to indigenous soil bacteria during the bioremediation process.

**Figure 7:**
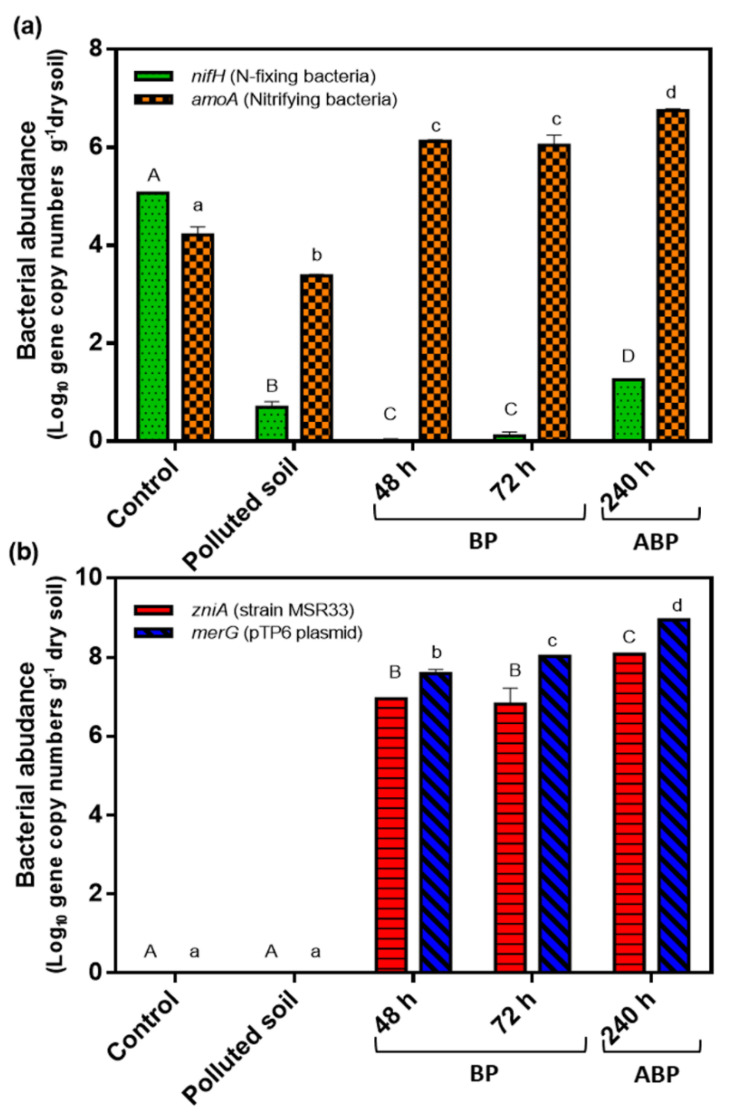
Effects of mercury (II) (20 ppm) and mercury bioremediation on gene copy numbers of *nifH*, AOB *amoA*, *zniA*, and *merG* in agricultural soils. (**a**), Copy numbers of nitrogen cycle genes (*nifH* and AOB *amoA*). (**b**), Copy numbers of strain MSR33 tracking *zniA* gene and of plasmid pTP6 tracking *merG* gene. Gene copy numbers were expressed in Log_10_. Control: metagenomic DNA of non-polluted soil; Polluted soil: metagenomic DNA of mercury-polluted soil (exposed to mercury (II) (20 ppm) for 1 week; 48 and 72 h: metagenomic DNA of soil samples withdrawn from rotary drum bioreactor (RDB) during bioremediation process (BP); 240 h: metagenomic DNA of soil samples withdrawn from RDB 1 week after bioremediation processes (ABP). Assays were performed in triplicate; bars indicate the standard deviation. Significant differences were analysed by one-way ANOVA followed by LSD Fisher test. Means with different letters indicate significant differences (*p* < 0.05). Capital letters indicate significant differences for *nifH* (**a**) and *zniA* (**b**) genes, and lowercase letters indicate significant differences for AOB *amoA* (**a**) and *merG* (**b**) genes.

To evaluate the effects of mercury and mercury bioremediation on the nitrogen cycle of agricultural soil, genes associated to nitrogen fixation (*nifH*) and nitrification (AOB *amoA*) were studied by absolute qPCR in soil (Figure 7).

The presence of mercury in agricultural soils strongly decreased the copy number of the *nifH* gene (5 copies) compared to control soil (1.14 × 10^5^ copies). During the bioremediation process (48 and 72 h), a significant decrease in the *nifH* gene copies was observed. At 240 h after the bioremediation process, a significant increase in copy number of *nifH* gene was observed (1 × 10^3^ copies). Mercury pollution decreased the copy number of AOB *amoA* gene in soils (2.3 × 10^3^ copies) compared to non-polluted control soil (1.6 × 10^4^ copies) (Figure 7). During the bioremediation process, a high increase in copy number of AOB *amoA* gene was observed (1.3 × 10^6^ copies). The highest levels of the AOB *amoA* gene were observed at 240 h after the bioremediation process (5.6 × 10^6^ copies).

During the bioremediation process of mercury-polluted soils, strain MSR33 was tracked through qPCR analysis of the *zniA* gene. The *zniA* gene is present in a single copy in the chromid of strain MSR33 and encodes a heavy metal cation pump. The plasmid pTP6 was tracked using the *merG* gene. The broad-spectrum mercury resistance *merG* gene is present in a single copy in plasmid pTP6 and encodes a permease for phenylmercury. The copy number of *zniA* gene was detected only during the soil bioremediation at 48 and 72 h and after the bioremediation process (240 h). The *zniA* gene was not detected in non-polluted control soil and non-bioremediated mercury-polluted soil (Figure 7). No significant differences were observed between the copy number of the *zniA* gene during 48 and 72 h (8.7 × 10^6^ and 6.5 × 10^6^ copies, respectively). A significant increase in the copy number of the *zniA* gene was observed at 240 h after the bioremediation process (1.1 × 10^8^ copies). The *merG* gene showed a similar pattern to the *zniA* gene. The *merG* gene was only detected during the soil bioremediation process, at 48 and 72 h, and after the bioremediation process (240 h). The *merG* gene was not observed in the non-polluted control soil and non-bioremediated mercury-polluted soil (Figure 7). High levels of the *merG* gene were observed during the bioremediation process (48 and 72 h) (3.1 × 10^7^ and 1.0 × 10^8^ copies, respectively) and after the bioremediation process (240 h) (8.7 × 10^8^ copies).

## 4. Discussion

Bioremediation and phytoremediation are technologies of increasing application for the clean-up of polluted soils. Microbes play a crucial role in bioremediation and phytoremediation [60,61,62,63]. However, the bioremediation of mercury-polluted soil has been scarcely studied [31,41,42,43]. In this study, we established a novel ex situ bioremediation technology using bioaugmentation by *C. metallidurans* MSR33 in an RDB for mercury removal in polluted soils. Therefore, in this study, an acrylic RDB (20 L) with 8 internal lifters, equipped with a humidified air injection system and a gas trap for gaseous mercury, was designed and built (Figure 1). This bioremediation treatment was an effective technology for high mercury removal (up to 82% after 48 h) in agricultural soils polluted with mercury II (20–30 ppm).

The bioremediation by bioaugmentation with strain MSR33 of mercury-polluted agricultural soils in the RDB showed high mercury removal under diverse conditions. However, in all assays, a residual mercury concentration was observed after the bioremediation processes. Residual mercury in soil can be attributed to the binding of non-bioavailable Hg (II) to the organic matter. Mercury (II) interacts strongly with sulfur ligands of the organic matter [17,18,22,30]. In this study, the addition of thioglycolate resulted in the highest mercury removal in soil. The addition of thioglycolate (5 mM) increases mercury removal (10%), reaching the lowest residual mercury content in soil (~2.5 ppm). Thioglycolate increases the mercury bioavailability from soil organic matter through the formation of the dimercaptide RS-Hg-SR that is more susceptible to reduction [19,64]. Rojas et al. [19] reported the complete mercury removal by strain MSR33 of mercury-polluted aqueous solutions (20 and 30 ppm) in 2 h, using 250 mL flasks (50 mL cellular suspension) and high aeration rate (6 vvm) in the presence of thioglycolate.

Interestingly, a faster mercury removal was observed by MSR33 inoculation with 6 g cells kg^−1^ dry soil compared with 3 g cells kg^−1^ dry soil in the first 48 h, but both treatments reached similar mercury removal after 72 h (Figure 2). These results may indicate the dependence between the cellular concentration and mercury removal in the first 48 h. Thereafter, a lower mercury bioavailability decreased its removal, reaching a similar mercury reduction with both cellular concentrations. Similar results were observed by bioremediation using *Pseudomonas putida* PpY101/pSR134 in mercury-polluted water, which showed a dependence between the cellular concentration and the mercury removal rate [39]. Aerobic mercury bioremediation in a bioreactor with strain MSR33 showed a fast mercury removal (20 ppm) in polluted water, reaching almost complete mercury reduction after 24 h [22]. In the present study, the re-inoculation of strain MSR33 was evaluated to rule out the potential inhibition of this strain and to increase the mercury removal during the bioremediation of mercury-polluted soil in the RDB. Additional cell re-inoculation at 48 h did not influence soil bioremediation, suggesting the dependency of strain MSR33 on mercury bioavailability for soil bioremediation.

Notably, a high mercury concentration (30 ppm) in soil did not negatively affect the removal of mercury by strain MSR33 (Figure 3). This result can be attributed to a higher mercury bioavailability that is susceptible to reduction by strain MSR33. In a previous study, we reported that strain MSR33 was capable of removing mercury (II) 24 ppm in polluted water [19]. However, mercury (II) 20 ppm inhibited the growth and respiratory rate of strain MSR33 in liquid medium under aerobic conditions; the inhibition was reversed after 5 h [22]. Mercury-polluted soil may dampen the harmful effects of high mercury concentration on the bioremediation by strain MSR33.

In this study, mercury (II) exposure reduced the number of total heterotrophic bacteria in agricultural soils, while an increase in Hg-tolerant bacteria was observed. These results indicate the susceptibility to mercury (II) of heterotrophic bacteria. The negative effects of mercury on bacteria have been widely described [17,19,65,66,67,68]. The pollution of mercury (4 weeks) changes the microbial community in agricultural soils, strongly decreasing bacteria of the Firmicutes phylum and increasing members of the Alphaproteobacteria class and the Planctomycetes phylum [13]. Short-term heavy metal pollution induces significant modifications in soil bacterial community structure [23]. The present study showed the presence of Hg-tolerant bacteria in non-polluted soil, whereas an increase of Hg-tolerant bacteria was observed 1 week after mercury pollution of the soil. The presence of bacteria with heavy metal tolerance in non-polluted soil has been reported, showing a significant increase of these bacteria in soils impacted with heavy metals [3]. Similarly, metagenomic analysis showed an increase of hydrocarbon-degrading bacteria in diesel-polluted soils [27]. During bioremediation, an increase of total heterotrophic and Hg-tolerant bacteria in agricultural soils was observed, which may be partly explained by the inoculation of strain MSR33 into the soil. Interestingly, bacterial abundance remained stable during 96 h of the bioremediation process.

In this report, mercury-resistant bacterial isolates from bioremediated mercury-polluted soils were identified as the gram-positive strains *Glutamicibacter* sp. SB1a, *Bacillus* sp. SB1b and *Planomicrobium* sp. SB2b, and the gram-negative strains *Bergeyella* sp. SB2a, *Brevundimonas* sp. SB3b and *Ochrobactrum* sp. SB4b (Figure 5). The isolation of mercury-tolerant bacteria (2–20 ppm) from diverse polluted environments have been described [69,70,71,72,73]. Our study is the first report describing mercury-resistant strains from the *Glutamicibacter*, *Planomicrobium* and *Bergeyella* genera. Nevertheless, heavy metal resistance in *Glutamicibacter* and *Planomicrobium* strains were described [74,75,76]. Mercury-tolerant *Bacillus* was isolated from mercury-contaminated soils, water, sediments, and High-Arctic snow and freshwater [63,69,71,73,77,78,79]. Mercury-tolerant *Ochrobactrum* strains were isolated from hydroelectric dam sediment and *Porcellio scaber* gut [70,71]. Mercury-tolerant strains of the *Brevundimonas* genus, including the mercury-resistant *Brevundimonas* sp. strains HgP1 and HgP2, were isolated from agricultural soil and gold mines [13,72]. In our study, selective pressure by mercury and the bioremediation process favored the increase of infrequent Hg-tolerant bacteria. This is in accordance with an increase of hydrocarbon-degrading bacteria in the soil after hydrocarbon pollution and bioremediation reported by Fuentes et al. [27]. Further studies are required for a deeper understanding of the effects of mercury pollution and bioremediation on bacterial communities in agricultural soils.

The presence of the *merG* gene in native Hg-tolerant strains was observed in this report. *Glutamicibacter* sp. SB1a, *Brevundimonas* sp. SB3b, and *Ochrobactrum* sp. SB4b contain the *merG* gene (Figure 6). The *merG* gene encodes for a periplasmic protein involved in cell permeability of phenylmercury, which is part of the broad-spectrum mercury resistance and is present in a single copy in plasmid pTP6 [19]. The presence of the *merG* gene in the *mer* operon showed a lower frequency (1.8% in 272 bacterial and archaeal *mer* operons analysed) compared to other *mer* genes encoding narrow-spectrum Hg resistance such as the *merA* gene [80]. The presence of the *merG* gene in native bacteria suggests the horizontal gene transfer (HGT) of the plasmid pTP6 from strain MSR33 to native strains in soil. In our study, the results suggest that strain MSR33 acts as a mercury resistance gene donor to native gram-positive and gram-negative bacteria such as *Glutamicibacter* sp. SB1a (Actinobacteria), *Brevundimonas* sp. SB3b, and *Ochrobactrum* sp. SB4b (Proteobacteria), favoring a bioremediation process mediated by strain MSR33 and native bacteria adapted to mercury-polluted soil. Therefore, the presence of the plasmid pTP6 in native strains should be further studied. The HGT is an important mechanism for increasing the degradative traits of microbial communities [61]. The HGT results from conjugation, transformation, or transduction, wherein the conjugation is the most important mechanism [81]. Plasmid-mediated genetic variation enables bacteria to respond promptly to challenges, such as the presence of antibiotics, heavy metals, and xenobiotic compounds [82]. The plasmid pTP6 was captured from contaminated sediment slurry in River Nura (Kazakhstan) by *Cupriavidus necator* JMP228 [83]. The horizontal transfer of IncP-1 plasmids closely related to plasmid pTP6 from the Proteobacteria phylum to Actinobacteria, Firmicutes, and Bacteroidetes phyla has been reported [84,85]. Inter-gram plasmid transfer of IncP-1 is a frequent phenomenon in soil [86]. Plasmid-mediated bioaugmentation has been proven to be effective for the clean-up of soils contaminated with heavy metals and organic compounds [87,88]. The broad-spectrum mercury resistance genes are part of the transposon Tn50580 in plasmid pTP6 [19,34,83]. Therefore, a transposon-mediated transfer of mercury resistance genes from strain MSR33 to native bacteria may occur. The transposon-mediated in situ transfer of the *mer* genes from introduced microbes to autochthonous bacteria has been recently described as an innovative technology for bioremediation [89].

In this study, the effects of mercury pollution and bioremediation by strain MSR33 in an RDB on nitrogen cycle microorganisms in agricultural soils were determined (Figure 7). The copy number of *nifH* gene (nitrogen fixers) and AOB *amoA* (nitrifying bacteria) gene quantified in non-polluted agricultural soil in this study is similar to gene levels described for agricultural soils [6,90]. Mercury pollution caused a decrease of the *nifH* gene (~4 orders of magnitude) and the AOB *amoA* gene (~1 order of magnitude), indicating the susceptibility to mercury of nitrogen-fixing and nitrifying bacteria communities in the soils. Exposure to heavy metals including mercury and pesticides induces alterations in the bacterial communities of agricultural soils [2,8,27,30,91]. In contrast, no significant differences in copy number of AOB *amoA* gene in soils after exposure to mercury (up to 200 ppm) have also been reported, however, the AOB *amoA* gene was quantified eight weeks after the mercury pollution event [90].

The bioremediation by strain MSR33 of mercury-polluted soils in the RDB decreased the copy numbers of the *nifH* gene, whereas the AOB *amoA* gene showed an increase during bioremediation, reaching even higher values than in non-polluted soil (Figure 7). This change in the microbial dynamics could be attributed to three factors: (i) mercury bioremediation process, (ii) increase of oxygen during the bioremediation, and (iii) the presence of exogenous nitrogen supplied by the GBC medium used for MSR33 growth. Firstly, it has been reported that bioremediation processes affect the dynamics of microbial communities [27,57]. Bioaugmentation by *Pseudomonas* sp. MHP41 of simazine-polluted agricultural soils changes the bacterial communities, increasing Acidobacteria and Planctomycetes phyla [57]. Hydrocarbon soil bioremediation showed changes in microbial community structure, observing a bloom of a specific bacterium present in low abundance before the pollution, and decreasing bacterial diversity and richness [27]. Secondly, in this study, the agricultural soil bioremediation process was performed in presence of oxygen in the RDB. Oxygen may damage the Fe-S groups of the nitrogenase reductase enzyme, inhibiting nitrogen-fixing diazotrophic bacteria [92]. On the other side, the presence of oxygen favors nitrification and increases nitrifying bacteria in agricultural soils [93]. Finally, the observed changes in copy number of the *nifH* and AOB *amoA* genes may be associated to external nitrogen sources. The addition of MSR33 cells grown in GBC medium that contains NH_4_Cl could affect the copy number of the *nifH* and AOB *amoA* genes during the bioremediation process. The incorporation of nitrogen in soils affects the structure of nitrogen cycle microorganisms [2,6]. The addition of urea stimulates the nitrogen fixation (*nifH*), nitrification (AOB *amoA*) and denitrification (*nirS*, *nirK*) bacteria in Antarctic soils [94]. The application of urea and compost in agricultural soils decreases the nitrogen-fixing microorganisms and increases the number of nitrifying microorganisms [6].

Interestingly, significant differences in the *nifH* and the AOB *amoA* genes levels were observed one week after bioremediation of mercury-polluted soils, showing an increase in the number of nitrogen-fixing and nitrifying bacteria (Figure 7). The copy number of the *nifH* gene showed a significant increase after bioremediation. The removal of mercury and the decrease in aeration could contribute to an increase of nitrogen-fixing bacteria, due to the absence of toxic effects caused by mercury and oxygen. The restoration of nitrogen-fixing bacterial populations could contribute to an increase in nitrifying bacteria, indicating the return of nitrogen cycle to natural conditions. These results indicate a positive effect of bioremediation of mercury-polluted agricultural soil on biological soil parameters associated with the nitrogen cycle. Nitrogen cycle-associated processes are performed by several microbial taxa. Within this study, only part of the microbial communities was evaluated. In this study, nitrifying archaea and denitrifying microorganisms were not studied, which may play an important role in nitrogen cycle of agricultural soils [2,5,6,8,22,95]. Further studies are required to understand the nitrogen cycle microbial dynamics during bioremediation by strain MSR33 of mercury-polluted agricultural soils.

This study showed that strain MSR33 inoculated during the bioremediation process was maintained during and after the treatment. An increase of the *zniA* (strain MSR33) and the *merG* (plasmid pTP6) genes at the end of the bioremediation process was observed, which is in agreement with the higher levels of Hg-tolerant bacteria (Figure 4 and Figure 7). It is well known in bioremediation processes that after the addition of a single bacterium or bacterial consortium into the soil, their abundance in soil decrease after some weeks, concomitant with an increase of native bacterial taxa [27,96]. Notably, strain MSR33 showed an increase after bioremediation, suggesting its adaptation to the soil. The adaptation and colonization of *C. metallidurans* MSR33 in mercury-polluted soils support its use as a protective agent for soils exposed to heavy metal pollution.

**Figure 8:**
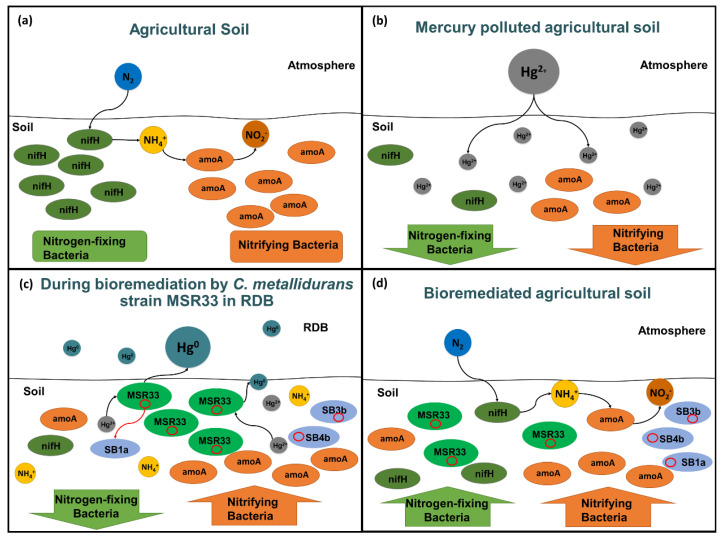
Overview of the effects of mercury and mercury bioremediation by *C. metallidurans* strain MSR33 of agricultural soil on nitrogen cycle microorganisms in rotary drum bioreactor (RDB). (**a**), Balance between nitrogen-fixing and nitrifying microorganisms in agricultural soil. (**b**), Inhibition of nitrogen-fixing and nitrifying microorganisms in agricultural soil exposed to mercury. (**c**), Imbalance between nitrogen-fixing and nitrifying microorganisms during mercury-polluted agricultural soil bioremediation by *C. metallidurans* strain MSR33 in RDB. (**d**), Re-establishment of nitrogen-fixing and nitrifying bacterial communities in mercury-remediated agricultural soil by *C. metallidurans* strain MSR33 in RDB. The plasmid pTP6 is represented in red circles on (**c**) and (**d**).

Figure 8 illustrates the effects of mercury pollution and bioremediation in agricultural soils. Mercury pollution disturbed bacterial communities in agricultural soil, inhibiting the native bacteria and specifically nitrogen cycle microorganisms. During bioremediation using strain MSR33 of mercury-polluted agricultural soil in an RDB, a decrease of nitrogen-fixing bacteria and an increase of nitrifying bacteria were observed. Strain MSR33 transferred mercury resistance genes probably through the plasmid pTP6 conjugation to indigenous bacteria. Strain MSR33 and native Hg-tolerant bacteria removed the bioavailable mercury from the agricultural soil. After bioremediation, strain MSR33 was maintained and nitrogen-fixing microbial communities were restored in the bioremediated soil, establishing a new microbial communities’ equilibrium.

Bioremediation by strain MSR33 in an RDB has a high potential for the treatment of soils polluted with heavy metals, such as mercury, cadmium, and copper, and persistent organic pollutants such as toluene [19,22,36,37]. This study suggests that bioremediation by bioaugmentation using bacteria (e.g., strain MSR33) in an RDB may be an attractive and innovative technology for the treatment of soils polluted with seed-coat dressing, pesticides, disinfectants, and pharmaceutical compounds, and soils impacted by mining towards a more sustainable development.

## 5. Conclusions

An innovative ex situ bioremediation process by *C. metallidurans* strain MSR33 of an agricultural soil polluted with Hg (II) (20 and 30 ppm) in a custom-made RDB was established, removing 82% of mercury in the presence of thioglycolate and 70% in absence of this agent. Mercury pollution affected soil nitrogen cycle bacteria, decreasing nitrogen-fixing bacteria and nitrifying bacteria. Specially nitrogen-fixing bacteria were highly sensitive to mercury pollution. During the bioremediation by strain MSR33 of mercury-polluted agricultural soil in the RDB, changes in nitrogen cycle communities were observed, increasing the nitrifying bacteria, but decreasing the nitrogen-fixing bacteria. During the bioremediation, strain MSR33 probably transfered the mercury resistance *merG* gene through the plasmid pTP6 into indigenous gram-positive and gram-negative bacteria, increasing their mercury resistance.

The bioremediation of mercury-polluted agricultural soil restored the nitrogen cycle, showing an increase of the nitrogen-fixing bacteria and the nitrifying bacteria. After bioremediation, strain MSR33 and native mercury-tolerant bacteria showed high levels in soil and may act as bioremediation catalysts in future mercury-pollution events.

## Figures and Tables

**Table 1:** Primers used in this study.

Primers	Sequence (5′-3′)	Gene Target	Size (pb)	Reference
27F	AGAGTTTGATCMTGGCTCAG	16S rRNA	1465	[53]
1492R	TACGGYTACCTTGTTACGACTT			
nifH-F-Rösch	AAAGGYGGWATCGGYAARTCCACCA	*nifH*	458	[54]
nifH-R-Rösch	TTGTTSGCSGCRTACATSGCCATCAT			
amoA-1F	GGGGTTTCTACTGGTGGT	AOB *amoA*	491	[55]
amoA-2R	CCCCTCKGSAAAGCCTTCTTC			
zniA_F	GGAAAGGCCTTCCTGGACAT	*zniA*	167	L. Rojas, personal communication
zniA_R	TCAACGCGGAGTTCTTCGTA			
merG_F	AGTACCGCAACGTTAGGCAT	*merG*	171	L. Rojas, personal communication
merG_R	ACCGCATTTGTACGCAAGAC			
